# Graphite nanoplatelet chemical cross-linking by elemental sulfur

**DOI:** 10.1186/1556-276X-8-94

**Published:** 2013-02-20

**Authors:** Gianfranco Carotenuto, Valentina Romeo, Sergio De Nicola, Luigi Nicolais

**Affiliations:** 1 Institute for Composite and Biomedical Materials, CNR. P.le Tecchio, 80, 80125, Naples, Italy; 2 Istituto Nazionale di Ottica, CNR, Via Campi Flegrei, 34, Pozzuoli 80078, Italy; 3 INFN Sezione di Napoli, Complesso Universitario di M.S. Angelo, via Cinthia, Naples 80126, Italy; 4 Department of Material Engineering and Production, University “Federico II” of Naples, P.le Tecchio, 80, Naples 80125, Italy

**Keywords:** Graphite nanoplatelets, Sulfur, Mechanical stabilization, Calorimetry

## Abstract

**Abstract:**

Graphite nanoplatelets (GNPs) react with elemental sulfur to provide a mechanically stable, spongy material characterized by good electrical conductivity and high surface development; such unique property combination makes these novel nanostructured materials very useful for applications in different technological fields. The carbon-sulfur reaction can be accurately investigated by thermal analysis (differential scanning calorimetry and thermogravimetric analysis) and energy-dispersive X-ray spectroscopy combined with scanning electron microscopy. The thermal treatment required for the formation of electrically conductive monosulfur connections among the GNP unities has been investigated.

**PACS:**

81.05.Ue, 81.05.Rm, 81.16.Be

## Background

Graphene, a single layer of carbon atoms arranged in a hexagonal network, is a 2D nanostructure with outstanding physical properties [1]. The successful isolation of graphene has had great interest for experimental investigations and has opened the way to a wide range of novel technological applications [1]. Recent studies have been directed toward using graphite nanoplatelets (GNPs) and graphene as a substrate to support nanostructures (e.g., quantum dots, metal catalysts, magnetic nanoparticles, etc.) because of their wide surface area, chemical stability, mechanical strength, and flexibility [2-4].

*sp*^2^ carbon nanoforms (e.g., fullerenes, CNTs, graphite nanoplatelets, and graphene) can be chemically cross-linked and polymerized by reaction with elemental sulfur. The resulting synthetic solid phases can be considered as a sort of three-dimensional polymers of sulfur and structurally complex carbon-based monomers. This carbon-sulfur chemical reaction may result in a certain importance in the preparation of novel bulky nanostructured materials [5]. For example, a highly spongy graphite-based material (graphite aerogels) can be prepared by drying concentrated GNP colloids, achieved by exfoliation of expanded graphite in nonpolar liquids with ultrasounds [6]. This novel material is quite fragile and has a measured apparent density of 0.5 g/cm^3^. A mechanical stabilization treatment is required to exploit this system in technological applications. The carbon-sulfur chemical reaction can be advantageously used for the mechanical stabilization of the very fragile spongy graphite material. The introduction of sulfur in this spongy graphite structure is quite simple since the sulfur molecules (S_8_) are soluble in nonpolar organic media (hydrocarbons, etc.), and it can be dissolved in the GNP colloid before the drying process. Then, the dry GNP-based material is heated at *ca*. 180°C to allow the sulfur molecules to open, producing sulfur bi-radicals (*∙*S_8_*∙*) which bridge the graphene layers of closed nanoplatelets [7]. In particular, the ring of sulfur molecule (S_8_) breaks at a temperature of *ca.* 169°C, producing linear sulfur bi-radical fragments, and such endothermal process is named as λ-transition [8]. The permanence of the system at temperatures above the λ-transition allows the polysulfur molecular chains (C-(S)_*n*_-C) to break successively and the generated sulfur radicals to react again with the edges of graphene sheets above to achieve a high density of monosulfur chemical cross-links (C-S-C) between them. The monosulfur bridges allow electron delocalization among the graphene sheets, and therefore, they represent a sort of electrical connections in the material. When the spongy graphite is devoted to technological applications in the electrical/electronic field (e.g., supercapacitor electrodes, battery cathodes, electrodes for electrolytic cells, etc.) [9], the presence of monosulfur bridges among the GNP unities is a very convenient characteristic. In addition, the material stiffness is related to the length of sulfur bridges, and monosulfur connections lead to a much more rigid and tough material.

## Methods

### Materials

Expandable graphite flakes (Asbury, Asbury, NJ, USA) underwent a thermal shock at 750°C for 3 min in a muffle furnace to produce expanded graphite (worm-like graphite). As-received elemental sulfur (99.9%, Sigma-Aldrich, Milan, Italy) was dissolved in octane (purum, Carlo Erba Reagents, Milan, Italy), and the expanded graphite filaments were added step by step to this sulfur solution during an ultrasound processing of the liquid system, done with a horn sonicator (20 KHz, 200 W, model UW2200, Bandelin Sonoplus, Berlin, Germany) at room temperature. The resulted expanded graphite filaments were completely converted to GNPs after ultrasound application. The final product was a sort of paste, which was dried in air at room temperature to produce a highly porous graphite/sulfur mixture, successively annealed in oven at 300°C in order to cross-link the material.

### DSC analysis

Dynamic calorimetric tests were carried out by a differential scanning calorimeter (DSC; Q2920, TA Instruments, New Castle, DE, USA). Measurements were performed under fluxing nitrogen at a rate of 10°C/min ranging from 20°C to 300°C.

### TGA analysis

Thermogravimetric analysis (TGA) was carried out using a thermobalance (Q5000, TA Instruments). In particular, the samples were heated from 30°C to 800°C at a rate of 10°C/min in fluxing air.

## Results and discussion

**Figure 1:**
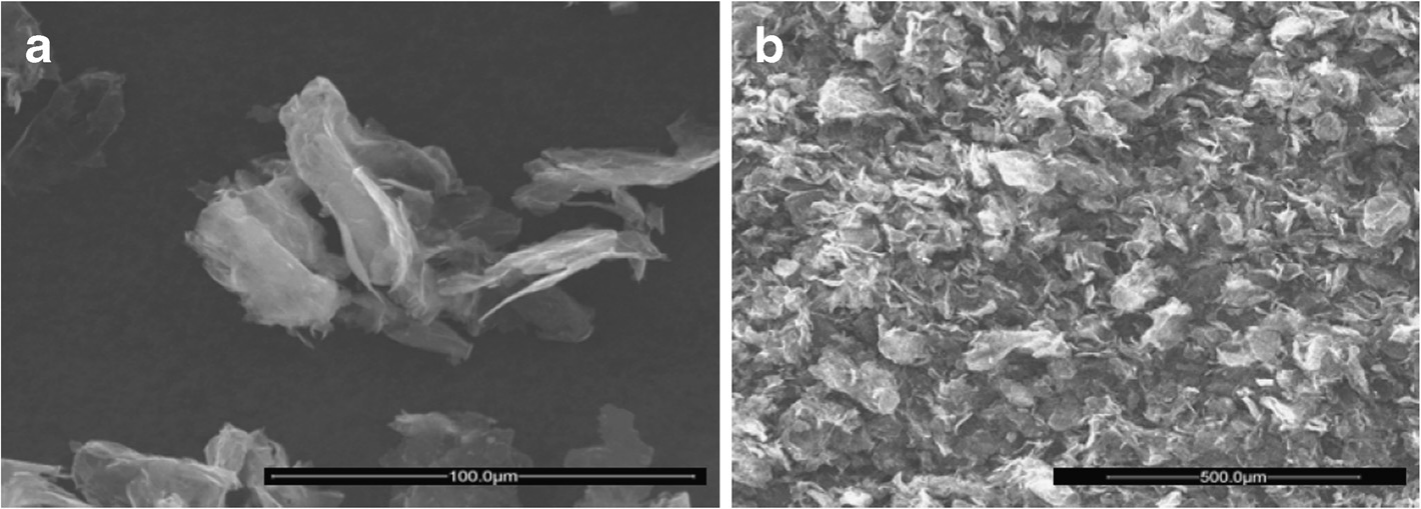
SEM micrographs showing the morphology of the graphite nanoplatelets (a) and the GNP aerogel (b).

The morphology of single GNP unities and their aerogels was investigated by scanning electron microscopy (SEM). The SEM micrograph of GNP is given in Figure 1a. The petal-shaped unities, shown in Figure 1a, have two main dimensions of *ca*. 80 μm and a thickness of only a few tens of nanometer. As visible in Figure 1b, these petal-like structures are randomly distributed in the aerogel bulk, and a very porous solid results.

**Figure 2:**
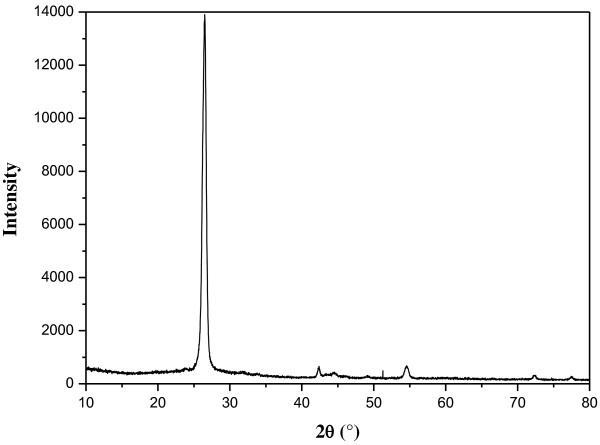
XRD diffractogram of the graphite nanoplatelet sample.

Figure 2 shows the X-ray diffraction (XRD) diffractogram of a graphite nanoplatelet sample. According to the Scherrer equation, the average GNP thickness is 15 nm.

**Figure 3:**
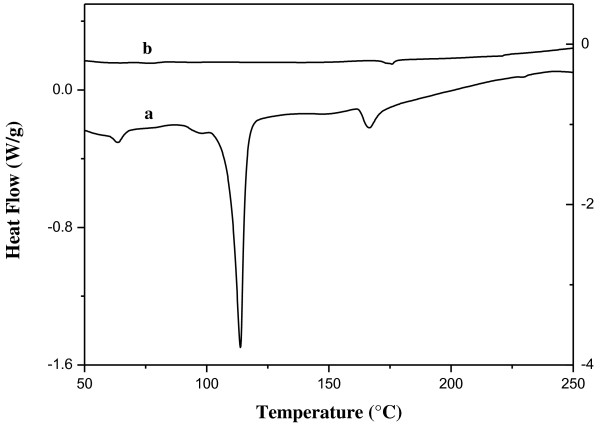
**DSC thermograms of the S/GNP system.** First (thermogram **a**) and second (thermogram **b**) heating run.

Graphite nanocrystals are much more chemically reactive than the ordinary graphite flakes; consequently, a number of graphite derivatives can be easily prepared using such nanoscopic graphite crystals as reactant (for example, graphite nanoplatelets can be quantitatively and quickly converted to graphite oxide by the Hummers method [10]). The free radical addition to the carbon-carbon double bond is a typical reaction involving benzene (C_6_H_6_) and other polycyclic aromatic compounds; as a consequence, graphene, fullerenes, carbon nanotubes, and other nanostructures based on the *sp*^2^ carbon could also give the same type of reaction. Therefore, the chemical cross-linking of graphite nanoplatelets could be based just on this type of reaction, but a bi-radical molecule should be used in order to graft simultaneously two GNP unities. Elemental sulfur is made of S_8_ rings, which is converted into a linear polymeric bi-radical molecules (·S-S_6_-S·) at a temperature of 160°C; such reaction is known as λ-transition. The λ-transition of elemental sulfur is an endothermic process which is clearly visible in a DSC thermogram [11]. In particular, the DSC thermogram of elemental sulfur contains three endothermic signals: (1) the α → β transition of the sulfur crystals at 98°C, (2) the melting of the β-crystals at 116°C, and (3) the λ-transition at 160°C (see Figure 3 (thermogram a) and Table 1).

**Table 1:** Thermodynamic properties of the S/GNP system obtained by DSC

***T***_**α → β**_	**Δ*H***_**α → β**_	***T***_**β**_	**Δ*H***_**β**_	***T***_**λ**_	**Δ*H***_**λ**_
**(°C)**	**(J/g)**	**(°C)**	**(J/g)**	**(°C)**	**(J/g)**
98	1.08	116	12.5	160	1.10

The isothermal annealing of the reactive sulfur/GNP system at temperatures higher than 160°C allows a more or less complete conversion of polysulfur bridges (C-S_8_-C) to monosulfur bridges (C-S-C) which are sort of electrical connections between the graphene planes because conjugation is possible through the sulfur atom. When the GNP-based aerogels are devoted to electrical applications (e.g., electrodes for batteries and supercapacitors, electrolysis cells, etc.), such type of chemical cross-linking results are extremely convenient.

The λ-transition is characterized by a clearly visible endothermic signal (the enthalpy change is 1.10 J/g), and it can be detected also in the DSC analysis of S/GNP mixtures (see Figure 3 (thermograms a and b)). Consequently, important information on the chemical interaction between sulfur and GNP can be obtained by DSC analysis.

In particular, the change of the S-S bond concentration (i.e., the [S-S]/[S-S]_0_ value) can be calculated by analyzing the change in the enthalpy variation of the λ-transition signal. In particular, the thermal treatment of the S/GNP systems significantly modifies the DSC thermogram: the melting peak of the β-sulfur at 116°C disappears, and the λ-transition peak results strongly decreased because the [S-S] is proportional to Δ*H* of the λ-transition. Such decrease of the λ-transition peak depends on time and temperature of the thermal annealing treatment. The fraction of reacted S-S bonds (*α*) is given by the following expression:

(1) $$\alpha =1-\left[S-S\right]/{\left[S-S\right]}_{0}=1-\mathrm{\Delta H}/\Delta H_{0}$$

**Figure 4:**
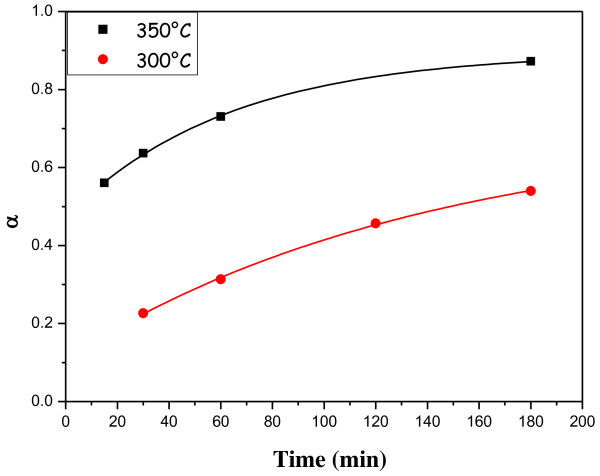
**Behavior of the reacted S-S bond fraction with time.** The experimental data points have been fitted by the exponential recovery law.

The temporal evolution of *α* at two different temperatures (300°C and 350°C) is shown in Figure 4. As visible, the experimental data are well described by an exponential recovery function (i.e., *α* = *a* − *b* × *e*^−*kt*^).

Such experimental behavior of the reaction conversion suggests the following three-step reaction mechanism:

$$\begin{array}{cc} S-S↔2S & \left(\text{slow}\right)\\ S+C=C\to S-C-C & \left(\text{fast}\right)\\ S+S-C-C\to S-C-C-S & \left(\text{fast}\right) \end{array}$$

The first reaction step involves the cleavage of the S-S bond with the formation of two sulfur radicals. This elemental reaction is reversible and has a slow specific rate. In the second elemental reaction, one of the two sulfur radicals is added to the carbon-carbon double bond with the formation of S-C bond and one carbon radical. Such reaction should have a fast rate because an unstable reactant (the sulfur radical) is involved. In the last elemental reaction, the carbon radical combines with the second sulfur radical with the formation of a new S-C bond. Also, this step should be very fast because the combination of two radicals is involved. The full reaction rate depends only on the slowest step which is characterized by a first-order kinetic; consequently, the rate expression is −*d*[S-S]/*dt* = *k*[S-S], which after integration provides an exponential recovery law (*α* = 1 − *e*^−*kt*^). Finally, according to the DSC analysis, the S/GNP chemical interaction is of the first kinetic order, and the involved mechanism is a direct reaction between the sulfur radicals generated at λ-transition and the *sp*^2^ carbon atoms located at the edges of the graphite nanocrystals.

In order to establish the temperature dependence of the reaction conversion, the rate constant of the reaction has been evaluated at different temperatures, giving for example the following values:

$$\begin{array}{l} k\left(573{}^{\circ}K\right)=7.22\cdot 10^{-3}\;{\text{min}}^{-1}\\ k\left(623{}^{\circ}K\right)=1.59\cdot 10^{-2}\;{\text{min}}^{-1} \end{array}$$

and these values have been used to evaluate the constants in the Arrhenius law:

(2) $$k=\mathrm{A\cdot}\text{exp}\left(-E_{a}/\mathrm{RT}\right)=136.76\cdot \text{exp}\left(-5644.0/T\right)$$

**Figure 5:**
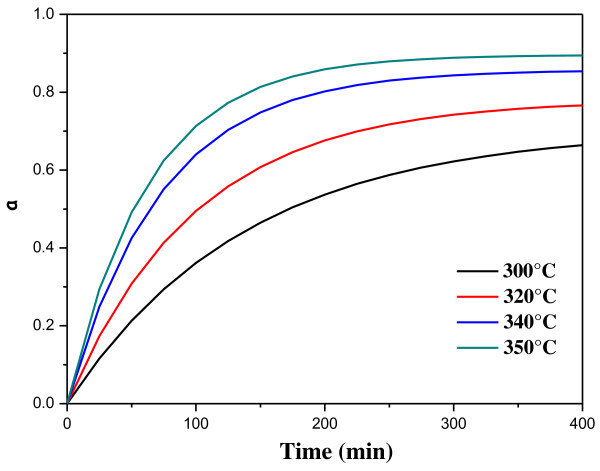
**Theoretical behavior of the time dependence of *α *at different temperatures.**

In particular, the activation energy of the reaction (46.9 kJ/mol) is in the same order of magnitude as a chemical bond (the S-S bond energy is *ca*. 213 kJ/mol). The behavior of the reaction conversion (*α*) under conditions different from that experimentally evaluated can be obtained by a simulation (the temperature values can be both interpolated or extrapolated). In Figure 5, the following expression has been used: *α* = *α*_max_ × [1-exp(−*kt*)] with *α*_max_ = −0.454 + 3.86 × 10^−3^ × *T*(°C) (a linear behavior has been assumed for the *α*_max_). As visible in Figure 5, a conversion degree close to 100%, which corresponds to a complete formation of monosulfur bridges (C-S-C), is possible only at a temperature higher than 350°C for a time period longer than 300 min.

**Figure 6:**
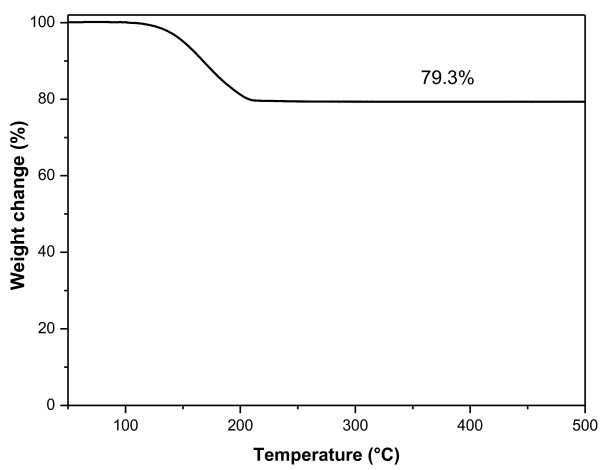
TGA thermogram of S/GNP mixture (50% by weight of sulfur).

The S/GNP chemical interaction was also investigated by thermogravimetric analysis. In particular, during the heating run (at 10°C/min) of a S/GNP sample (50% by weight of sulfur), some of the elemental sulfur reacts with carbon and bonds at GNP edges. In fact, such sulfur fraction cannot evaporate also at temperatures higher than the pure sulfur boiling point (444°C), and a residual sulfur content (*ca*. 30% by weight) results in the material, as visible in the TGA thermogram shown in Figure 6.

**Figure 7:**
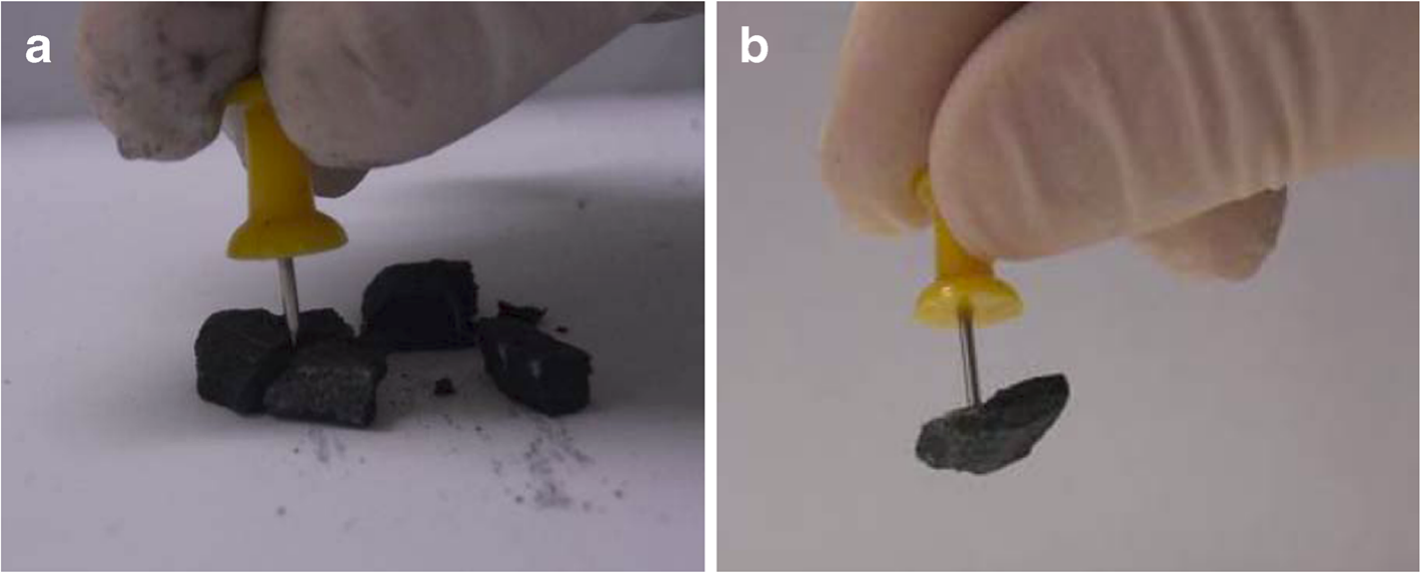
Fragile structure of the GNP aerogel (a) results mechanically stabilized by treatment with elemental sulfur (b).

It has been found that mechanically resistant GNP aerogels resulted after a cross-linking treatment with elemental sulfur at 350°C for 3 h (see Figure 7). A large number of electrically conductive monosulfur bridges should be generated in these conditions, and a good electrical conductor results (with resistivity of 3 Ω cm).

## Conclusions

In conclusion, the graphite nanoplatelets are very useful nanostructured materials that can be easily prepared by the oxidation-expansion-exfoliation process. GNP-based aerogels can be simply obtained by drying the concentrated GNP colloidal suspensions, and the introduction of elemental sulfur in the GNP aerogel followed by an adequate thermal annealing treatment allows a very good mechanical stabilization of the material by formation of monosulfur and polysulfur bridges between adjacent GNP unities.

## Competing interests

The authors declare that they have no competing interests.

## Authors’ contributions

VR carried out the experiments and prepared the samples. GC conceived of the experimental design and carried out the kinetic analysis. SDN developed the theoretical model and co-wrote the paper. LN participated in the design of the experiment and coordination. All authors read and approved the final manuscript.

## Authors’ information

GC is a senior researcher of the Italian National Research Council, Institute for Composite and Biomedical Materials. His present research interests are in the field of advanced functional materials based on polymer-embedded inorganic nanostructures. In particular, his activity concerns the development of new chemical routes for the controlled synthesis of metal and semiconductor clusters in polymeric matrices, the fabrication of devices based on properties of nanoscopic objects (e.g., luminescence of quantum dots, tunable surface plasmon absorption of nano-sized noble metal alloys, etc.), and the investigation of mechanisms involved in atomic and molecular cluster formation in polymeric media (nucleation, growth, aggregation, etc.) by optical and luminescence spectroscopy. He has authored 150 research articles published in international journals, ten patents, and many conference papers. He is the editor of two Wiley books devoted to metal-polymer nanocomposites and is a member of the editorial board of different scientific journals.

VR received her PhD in chemical engineering at the University of Salerno-Italy. During her PhD study, she spent a research period at the Institute of Polymer and Fibers in Moldal (Goteborg-Sweden), where she studied the effect of nanoparticle addition on the nanofibers obtained with electrospinning technique. She was a consulting engineer at the Department of Chemical and Food Engineering - University of Salerno for the project ‘Innovative technologies for production of new nanocomposite and carbon nanotubes.’ Currently, she is a scientific consultant of the Italian National Research Council, Institute for Composite and Biomedical Materials, for the project ‘AUTOSUPERCAP’ (Development of high energy/high power density supercapacitors for automotive applications). Her research interests include the preparation of nanostructure carbon materials.

SDN received his BS degree in physics from the University of Naples “Federico II”, Italy, in 1982. From 1983 to 1987, he was a system analyst at Elettronica (Rome) and Alenia (Naples). Since 1988, he has been a senior researcher of the Institute of Cybernetics “E. Caianiello” of the National Council of Research (CNR). Since 2010, he has been a member of the optical staff of the Italian National Institute of Optics (INO-CNR). He has been a scientific coordinator of the research project ‘Imaging Techniques for Studying and Analyzing Microstructured Materials’ of the CNR Department of Material and Devices. He has been a coordinator of the research unit based at the Institute of Cybernetics in the framework of the Italian National Research FIRB programme: Photonic Microdevices in Lithium Niobate. He has contributed to about 300 technical papers in peer-reviewed international journals, book chapters, and conference proceedings. He has served in program committees of several international conferences and has been a referee for various journals in the field of optics and theoretical physics. His research interests include the development and applications of non-destructive methods for material evaluation, optical metrology, theoretical modeling of laser beam propagation in heterogeneous media and nanostructured composites, nonlinear optical effects in cavity, quantum optics, laser-plasma interactions, spectroscopic techniques for nanostructured material, and development of quantum-like models in mesoscopic physics.

LN is President of the National Research Council of Italy, professor emeritus at the University of Naples “Federico II”, and adjunct professor at the Universities of Connecticut in Storrs and Washington in Seattle. He has a prepost of the Schools of Science, Engineering, and Architecture of the University of Naples “Federico II”. He is the author of more than 500 papers in scientific journals and 35 patents and is also the editor of 15 books. He is a member of the editorial boards of many scientific journals. He was awarded the SAMPE (Society for the Advancement of Materials Technology) honor certificate, the ‘G. Dorsi’ and ‘Scanno’ prizes, and the gold medal of the Academy of the Forty. LN significantly contributed to the development of knowledge in the field of composite materials, rheology, energy and mass diffusion through polymers, and materials for biomedical application.
